# Supplementary material for: High-Throughput Phenotypic Characterization and Diversity Analysis of Soybean Roots (Glycine max L.)
Source: Plants (Basel). 2022 Aug 2;11(15):2017. doi: 10.3390/plants11152017 (PMC9370148; doi:10.3390/plants11152017)
Supplement: Supplementary file 1 [file plants-11-02017-s001.zip › plants-1762121-supplementary.pdf]

Table S1. Soil physical properties.

Apparent density (Mg/m²)	pH	Ec (ds/m)	Soil composition (%)
0.3 ≧	5~7	≦ 1.2	Cocopeat 51.5; Peatmoss 10; Vermiculite 13; Perlite 15; Zeolite 10; Humic acid 0.1; Fertiliser 0.4

Table S2. Top and bottom 10 germplasms of each RMT. Each abbreviation indicated total length (LENGTH), surface area (SA), average diameter (DIAM), number of tip (NT), number of fork (NF), link average length (LAL), link average diameter (LAD), and link average branching angle (LABA), respectively.

Top 10 of RMT				Bottom of 10 of RMT			
Rank	IT No.	LENGTH (cm)	Origin	Rank	IT No.	LENGTH (cm)	Origin
1	208321	3228.24	JPN	1	162486	703.33	CHN
2	216313	3085.51	KOR	2	168187	730.5	JPN
3	216137	2985.57	KOR	3	162133	836.83	JPN
4	285979	2849.86	KOR	4	158022	880.6	JPN
5	312160	2792.67	KOR	5	160839	900.35	PRK
6	262976	2728.39	KOR	6	161747	925.22	CHN
7	263099	2723.51	KOR	7	161880	932.64	PRK
8	289377	2693.75	KOR	8	161833	965.91	JPN
9	289325	2673.7	KOR	9	162431	973.37	CHN
10	162339	2649.26	PRK	10	161579	976.88	CHN
Rank	IT No.	SA (cm)	Origin	Rank	IT No.	SA (cm)	Origin
1	208321	524.13	JPN	1	162486	124.15	CHN
2	216313	508.39	KOR	2	161747	127.66	CHN
3	285979	476.6	KOR	3	168187	131.04	JPN
4	177193	466.83	KOR	4	158022	145.79	JPN
5	262976	454.23	KOR	5	162133	149.57	JPN
6	185311	449.9	JPN	6	319450	153.89	JPN
7	263250	445.02	KOR	7	161579	156.37	CHN
8	289328	443.15	KOR	8	161833	156.52	JPN
9	289325	439.16	KOR	9	322902	158.7	KOR
10	162343	438.41	PRK	10	158058	159.77	JPN
Rank	IT No.	DIAM (cm)	Origin	Rank	IT No.	DIAM (cm)	Origin
1	242607	0.68	KOR	1	284095	0.41	JPN
2	160851	0.65	PRK	2	216137	0.42	KOR
3	162614	0.65	PRK	3	216138	0.43	KOR
4	162021	0.65	JPN	4	319452	0.43	JPN
5	160839	0.65	PRK	5	262888	0.43	JPN
6	162406	0.64	CHN	6	219522	0.44	KOR
7	161880	0.64	PRK	7	161747	0.44	CHN
8	237550	0.63	KOR	8	161746	0.45	CHN
9	269586	0.63	JPN	9	212802	0.45	KOR
10	242608	0.63	KOR	10	219511	0.45	KOR
Rank	IT No.	NT (Number)	Origin	Rank	IT No.	NT (Number)	Origin
1	263099	3305.67	KOR	1	162486	622.67	CHN
2	177192	3249.33	KOR	2	162133	624.67	JPN
3	185276	3213.33	JPN	3	162614	653	PRK
4	216313	3192.67	KOR	4	160839	766.67	PRK
5	263100	3167.33	KOR	5	158022	867.33	JPN
6	289325	3134	KOR	6	162508	898.67	CHN

7	216137	3097	KOR	7	162514	908	CHN
8	216139	3012.67	KOR	8	231347	913	KOR
9	168180	2990	CHN	9	224891	918.67	JPN
10	263098	2974	KOR	10	162371	921.33	CHN
**Rank**	**IT No.**	**NF (Number)**	**Origin**	**Rank**	**IT No.**	**NF (Number)**	**Origin**
1	216313	11638.33	KOR	1	162486	1180	CHN
2	208321	11289.67	JPN	2	168187	1342.67	JPN
3	285979	11099	KOR	3	161747	1638.33	CHN
4	185311	9922	JPN	4	162133	1848.33	JPN
5	162039	9383	JPN	5	161833	2100	JPN
6	177193	9372.33	KOR	6	161579	2114.67	CHN
7	285978	9283	KOR	7	158022	2138	JPN
8	289325	9066	KOR	8	161740	2154	CHN
9	289377	8977	KOR	9	162139	2201.67	PRK
10	289274	8948.67	KOR	10	319450	2239.67	JPN
**Rank**	**IT No.**	**LAL (Number)**	**Origin**	**Rank**	**IT No.**	**LAL (Number)**	**Origin**
1	160933	0.38	PRK	1	285978	0.15	KOR
2	322902	0.34	KOR	2	285979	0.16	KOR
3	162486	0.34	CHN	3	185311	0.16	JPN
4	158058	0.33	JPN	4	160955	0.16	JPN
5	161579	0.31	CHN	5	224517	0.16	KOR
6	267358	0.3	JPN	6	162150	0.16	PRK
7	162106	0.3	PRK	7	173051	0.16	JPN
8	162192	0.3	CHN	8	269645	0.16	KOR
9	161740	0.3	CHN	9	162256	0.16	PRK
10	161833	0.3	JPN	10	160812	0.16	PRK
**Rank**	**IT No.**	**LAD (Number)**	**Origin**	**Rank**	**IT No.**	**LAD (Number)**	**Origin**
1	242607	0.73	KOR	1	284095	0.39	JPN
2	160851	0.73	PRK	2	161747	0.41	CHN
3	289329	0.7	KOR	3	216137	0.42	KOR
4	160839	0.7	PRK	4	216138	0.43	KOR
5	162614	0.69	PRK	5	262888	0.43	JPN
6	162021	0.69	JPN	6	319452	0.45	JPN
7	162320	0.67	PRK	7	212802	0.45	KOR
8	143185	0.67	JPN	8	161746	0.46	CHN
9	185290	0.67	JPN	9	231346	0.46	KOR
10	269607	0.67	KOR	10	219522	0.46	KOR
**Rank**	**IT No.**	**LABA (Degree)**	**Origin**	**Rank**	**IT No.**	**LABA (Degree)**	**Origin**
1	224894	82.51	JPN	1	162458	9.15	PRK
2	161878	71.7	PRK	2	162074	9.91	JPN
3	161623	58.85	CHN	3	162526	13.07	CHN
4	229083	58.78	KOR	4	162339	17.05	PRK
5	263095	57.68	KOR	5	162133	17.8	JPN
6	185311	57.2	JPN	6	224516	17.87	KOR
7	185290	57.11	JPN	7	319451	18.01	JPN
8	285978	57.03	KOR	8	242607	18.01	KOR
9	185237	56.95	JPN	9	280127	18.12	JPN
10	162711	56.92	JPN	10	162579	18.2	CHN

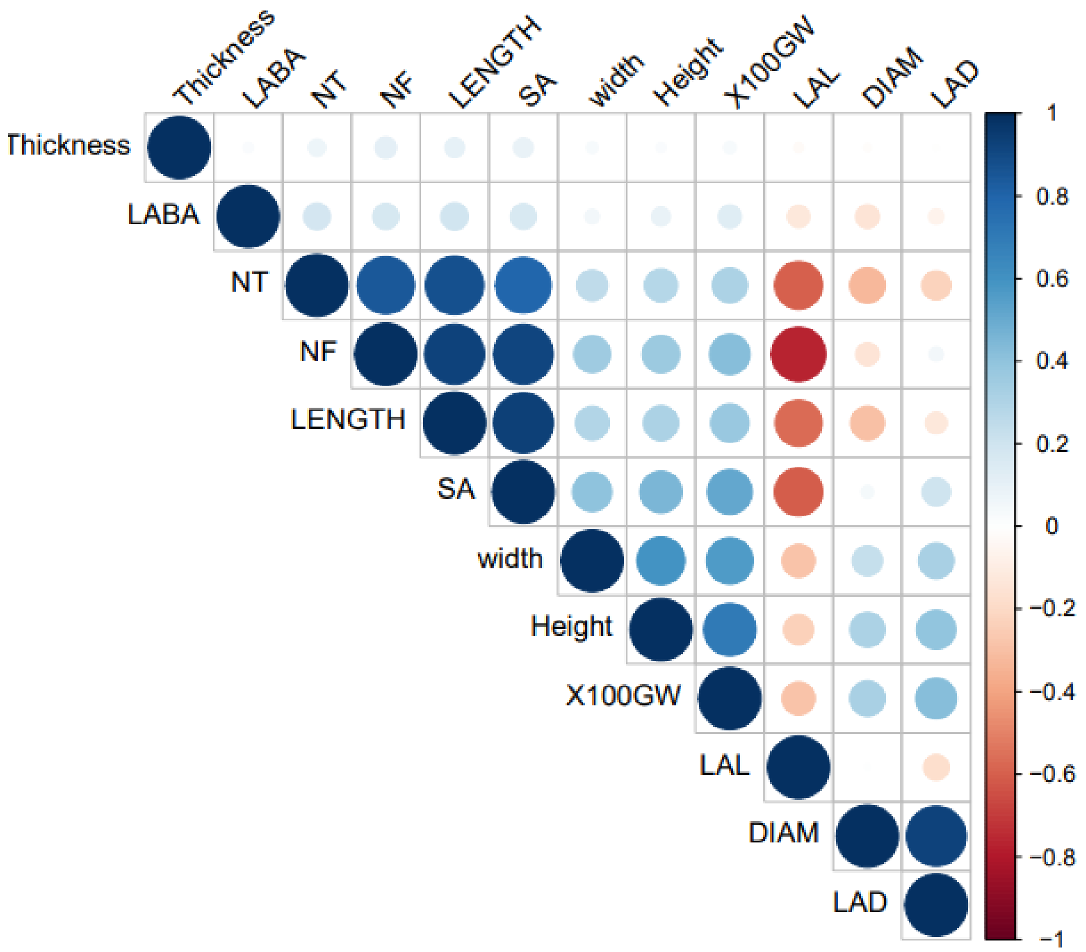

Figure S1. Correlation analysis of eight root morphological traits with seed morphological traits. Width (width of seed); Height (height of seed); 100GW (100-seed weight).
